# Combined use of the automated breast volume scanner and the US elastography for the differentiation of benign from malignant lesions of the breast

**DOI:** 10.1186/1471-2407-14-798

**Published:** 2014-11-03

**Authors:** Chaoli Xu, Shuping Wei, Yingdong Xie, Xiaoxiang Guan, Ninghua Fu, Pengfei Huang, Bin Yang

**Affiliations:** Department of Ultrasound Diagnostics, Jinling Hospital, Nanjing University School of Medicine, 305 East Zhongshan Road, Nanjing, Jiangsu 210002 China; Departments of Medical Oncology, Jinling Hospital, Nanjing University School of Medicine, Nanjing, 210002 Jiangsu, China

**Keywords:** Automated breast volume scanner, ABVS, US elastography, UE, *Kappa* statistics, Breast cancer

## Abstract

**Background:**

Automated breast volume scanner (ABVS) and US elastography (UE) have been useful for the differentiation of benign and malignant lesions. However, combining these two methods applied in diagnosis of breast lesions has not yet been reported. The aim of this study is to analyze the inter-examiner reliability of ABVS and UE, and compare diagnostic performance among ABVS, UE, and the combination of these two methods.

**Methods:**

Forty-one patients (forty-six lesions) underwent both ABVS and UE examinations. ABVS images were acquired by medial and lateral scans for each breast and classified a BI-RADS category based on the distribution, size, shape, echogenicity and microcalcification of the lesions. UE images were assigned an elasticity score according to the distribution of strain induced by light compression. *Kappa* statistics was used to examine the reproducibility between examiners with ABVS and UE, and the concordance between pathology and ABVS, UE, and the combination of these two methods. χ^*2*^ test was used to compare diagnostic performance among these three methods.

Two examiners blinded to the patients’ history evaluated the results of breast imaging independently.

**Results:**

Inter-examiner reliability with ABVS (*κ* = 0.62, 95% confidence interval (CI): 0.44-0.80) and UE (*κ* = 0.65, 95% CI: 0.48-0.82) was substantial. With respect to the pathology results, the inter-rater coefficient of concordance was *κ* = 0.81 (95% CI: 0.64-0.98) for ABVS, *κ* = 0.77 (95% CI: 0.58-0.96) for UE, and *κ* = 0.90 (95% CI: 0.77-1.00) for combination of ABVS and UE. Examiner variability was reduced from UE to ABVS, and to the combination of ABVS with UE.

The diagnostic accuracy, sensitivity, and specificity for the combination of ABVS and UE were 95.7% (95%CI: 84.0-99.2), 100% (95% CI: 85.9-100), and 87.5% (95% CI: 60.4-97.8), respectively. When comparing, the diagnostic performance of ABVS combined with UE was better than, or at least equal to, that of ABVS (accuracy 91.3% (95% CI: 78.3-97.2), sensitivity 100% (95% CI: 85.0-1.00), specificity 77.8% (95% CI: 51.9-92.6)) or UE (accuracy 89.1% (95% CI: 75.6-95.9), sensitivity 96.4% (95% CI: 79.8-99.8), specificity 77.8% (95% CI: 51.9-92.6)) alone, though the improvement was no statistically significance.

**Conclusions:**

Both ABVS and UE demonstrated substantial inter-examiner reliability. With high diagnostic performance for differentiation of benign and malignant lesions in the breast, the combination of ABVS and UE are useful to improve the diagnostic accuracy and specificity.

## Background

Breast cancer occurs in millions worldwide with an increasing incidence. According to the American Cancer Society reported in 2013 [1], the incidence of breast cancer is the highest with a mortality the second among all cancers in the developed regions of the world such as the European and American countries, while relatively low, but rising in the developing regions. Detection and diagnosis of early stage tumors even microcarcinomas through innovation of diagnostic technologies may provide reliable and timely information for clinical treatment [2].

Ultrasonography (US) with the capability of evaluating breast tissue was first described nearly 60 years ago [3], and has undergone technical advancements, including Color Doppler, ABVS, and UE. Especially ABVS and UE, have become promising methods in detecting breast lesions.

ABVS is in its third decade [4]. It was initially designed to examine the whole breast with eight probes and a water tank, but limited by its low resolution [5–7]. With technological improvement, current ABVS is equipped with a 14 MHz transducer with the capability of scanning the whole breast automatically [8]. Consequently, the resolution of image is increased by providing better demonstration of breast anatomy and proper orientation. And the operator variability is reduced while the reproducibility is improved. Furthermore, it is time-saving, requiring only 10 min to scan a breast by a trained medical technologist [9]. This offers a direct and convenient method for specialists to make a diagnosis from images. However, without substantive breakthrough in diagnosis performance, its vital role of producing automatic, high-resolution whole breast imaging cannot replace handheld ultrasonography (HHUS). Therefore, it is undesirable for clinical practice in United States. FDA has recommended approval for use in screening of women with dense breast parenchyma because it is unsusceptible to breast density [10]. However, with its striking practical advantages mentioned above, ABVS is accepted by other countries, and its diagnostic performance was not inferior to HHUS [11–17].

Nevertheless, ABVS is out of its range when assessing lesions by stiffness. Instead, US elastography (UE), which was first described in 1990 [18], may compensate for this disadvantage. By measuring displacement (strain) within the tissue produced by compression [19], UE can evaluate the feature of lesions’ hardness providing additional information to distinguish benign from malignant masses with sensitivity of 78.0%-100% and specificity of 21.0%-98.5% [20]. Furthermore, UE would increase the sensitivity of B-mode sonography in detecting metastatic axillary lymph nodes [21] and distinguishing benign and malignant lesions associated with microcalcifications detected at screening mammography [22]. As for image acquisition, compressive force was required to be appropriate based on algorithm, which may affect the quality of elastogram [23].

The current study is designed to evaluate whether ABVS combined with UE would provide complementary information to the differentiation of benign and malignant lesions.

## Methods

### Patients

41 patients (46 lesions, ages 19–88 years, mean46 ± 1.6 years, 1 male and 40 female) underwent ABVS and UE at Jinling Hospital from October 2013 to April 2014 were retrospectively enrolled for the study. The diameter of lesions ranged 4.2-62 mm, with a mean 25 ± 2.3 mm. All 46 lesions (18 benign lesions and 28 malignant lesions) from above 41 patients had ultrasound-guided core needle biopsy to acquire their target breast tissue and then confirm their pathological type. A panel formalin-fixed paraffin-embedded breast tumor specimens was obtained from the archival resource of the Department of Pathology of Jinling Hospital. Patients without pathological results or with skin burst, sharp pain, poor compliance were excluded from the study. All patients signed informed consent before the ABVS examination, UE examination or ultrasound-guided core needle biopsy, and the study was approved by Ethics Committee of Jinling Hospital.

### Equipment and data acquisition

ABVS was performed by using ACUSON S2000 ABVS system (Siemens Medical Solutions, Mountain View, CA, USA) with a 14 MHz high-frequency linear transducer, which is capable of acquiring complete image of the breast (17 × 15 × 6 cm^3^, 318 two-dimensional slices) automatically in a single scan in approximately one minute. Examiners selected the most suitable settings for patients according to their breast size (A-D and DD cups), if the breasts were not full enough to contact with the compression paddle, ultrasound gel was used to expand contact area. Each breast was routinely scanned twice (medial and lateral). Patients were in supine position with slow and shallow breath and the arms above the head till the scan was completed. Images were sent to diagnostic workstation for reconstructing coronal 3D images.

UE was performed by using the same equipment as for ABVS. Examiners operated the probe (9 L4 liner transducer, 4-9 MHz) with light pressure that maintained contact with skin, and perpendicular to the lesions. The images were displayed with a scale from pink (softest component), to green (intermediate component), to red (hardest component). The compression was indicated to be just enough when the subcutaneous fat layer appeared as a mix of pink and green and the pectoralis muscle layer as a mixed of yellow and green. A region of interest (ROI) needed to be set to center the target lesion and around with the surrounding tissue like fat, muscle, and normal mammary glands. Patients were in supine position with breath holding. The real-time strain images were acquired after the compression.

### Images analysis and classification of lesions

#### ABVS images

Based on the characteristics of the lesions including the number of lesions, distribution, size, shape (smooth or irregular), echogenicity (hypoecho, isoecho, or hyperecho), and microcalcification, ABVS results were classified into five categories (0 = incomplete, needing additional assessment; 1 = normal; 2 = benign; 3 = probably benign; 4 = probably malignant; 5 = highly suggestive of malignancy) according to the American College of Radiologists Breast Imaging Reporting and Data System (ACR BI-RADS) [24]. In our study, benign lesions were considered to be BI-RADS category 1 to 3, and malignant lesions were category 4 to 5. Interpretation of the images was accomplished by two examiners independently who specialized in ultrasonography more than ten years.

#### UE images

In our study, almost all the lesions (one lesion was complex enchogenicity) were hypoechoic. We only compared the color mode in the lesions with surrounding breast tissue, assigning each image an elasticity score on a five-point scale. Generally, the higher share of blue color represent the harder lesion and the lower share of red color represent the softer lesion displayed in elasticity image. However, the color mode of Siemens free-hand elasticity software can be inversed as red indicating hard lesions whereas pink indicating soft lesions. Therefore, the scoring criteria were showed in Table 1. The score 1–3 were classified as benign, and score 4–5 classified as malignant. The interpretation of images was done in same fashion as mentioned above.

**Table 1:** **The scoring criteria of UE**

Score	Chromatic code	Possible lesions
**1**	Entirely pink	Prevalently elastic: prevalently the benign lesions
**2**	A mosaic pattern of purple mixed with a small amount of green	
**3**	A mosaic pattern of green mixed with a small amount of yellow	
**4**	Almost the entire lesion in yellow, but mixed with a small amount of red	Prevalently rigid: prevalently the malignant lesions
**5**	Both the lesion and surrounding area are red mixed with a small amount of yellow	

#### Statistical analysis

*Kappa* statistics was used to interpret the concordance between examiners with ABVS and UE, and the concordance between pathology and ABVS, UE, and the combination of these two methods. The values of *κ* <0 indicates no agreement, *κ* 0*–*0.20 slight, *κ* 0.21-0.40 fair, *κ* 0.41-0.60 moderate, *κ* 0.61-0.80 substantial, and *κ* 0.81-1.00 almost perfect agreement [25]. The accuracy, sensitivity, specificity, PPV and NPP were calculated, and *χ*^*2*^ test was used to compare diagnostic performance among these three methods. Statistical significance was assumed as *P* < 0.05 for all tests. The software package SPSS statistics version 16.0 (SPSS Inc, Chicago, USA) was used for the statistical analysis.

## Results

### ABVS

In our study, 46 lesions in 41 patients were evaluated by two examiners independently. According to examiner 1, none of the lesions was rated as BI-RADS 1, 7 lesions as BI-RADS 2, 8 lesions as BI-RADS 3, 19 lesions as BI-RADS 4 and 12 lesions as BI-RADS 5. Overall, 15 lesions were rated as benign and 31 lesions as malignant. As to examiner 2, none of the lesions was rated as BI-RADS 1, 7 lesions as BI-RADS 2, 7 lesions as BI-RADS 3, 25 lesions as BI-RADS 4 and 7 lesions as BI-RADS 5. In general, 14 lesions were rated as benign and 32 lesions as malignant. There was a substantial agreement (*κ* = 0.62, 95% CI: 0.44-0.80) between examiner 1 and examiner 2 (Table 2).

**Table 2:** ***Kappa*** **statistics of examiners with ABVS results**

ABVS		Examiner2					
		BI-RADS1	BI-RADS2	BI-RADS3	BI-RADS4	BI-RADS5	Total
	BI-RADS1	0	0	0	0	0	0
	BI-RADS2	0	5	2	0	0	7
	BI-RADS3	0	2	4	2	0	8
**Examiner1**	BI-RADS4	0	0	1	18	0	19
	BI-RADS5	0	0	0	5	7	12
	Total	0	7	7	25	7	46

*κ* = 0.62 (95% CI: 0.44-0.80), indicating the inter-examiner reliability reached a substantial agreement.

Looking closely at the results of ABVS, lesions of BI-RADS 4 and BI-RADS 5 were in major differences between examiner 1 and examiner 2. After discussion, examiners got consistent results (Table 3) for the purpose of better compared with the pathological category. *Kappa* statistics was used to analyze the agreement between final ABVS results and pathological findings, which reached an almost perfect agreement (*κ* = 0.810 (95% CI: 0.64-0.98) (Table 4).

**Table 3:** **The final results of ABVS, UE and pathology**

Examination	Lesions
ABVS	
BI-RADS1	0
BI-RADS2	5
BI-RADS3	9
BI-RADS4	20
BI-RADS5	12
UE	
Score1	0
Score2	8
Score3	7
Score4	25
Score5	6
Pathology	
Benign	18
Mammary dysplasia	6
Fibroadenoma	8
Intraductal papilloma	4
Malignant	28
Invasive ductal carcinoma	27
Invasive cribriform carcinoma	1

**Table 4:** ***Kappa*** **statistics of ABVS, UE, and ABVS + UE results with pathological findings**

Results		Pathology		
		Malignant	Benign	Total
**ABVS (** ***κ*** **= 0.81, 95%CI: 0.64-0.98)**	Malignant	28	4	32
	Benign	0	14	14
	total	28	18	46
**UE (** ***κ*** **= 0.77, 95%CI: 0.58-0.96)**	Malignant	27	4	31
	Benign	1	14	15
	total	28	18	46
**ABVS + UE (** ***κ*** **= 0.90,95% CI: 0.77-1.00)**	Malignant	30	2	32
	Benign	0	14	14
	Total	30	16	46

The inter-rater reliability coefficients of ABVS, UE and ABVS + UE were calculated.

### UE

Of the 46 lesions, examiner 1 graded none of the lesions with a score of 1, 9 lesions a score of 2, 7 lesions a score of 3, 26 lesions a score of 4, 4 lesions a score of 5. With respect to examiner 2, none of the lesions had a score of 1, 8 lesions had a score of 2, 11 lesions had a score of 3, 18 lesions had a score of 4, 9 lesions had a score of 5. There were 19 benign lesions and 27 malignant lesions determined by examiner 1 while 16 benign lesions and 30 malignant lesions were determined by examiner 2. The inter-examiner reliability obtained a substantial agreement (*κ* = 0.65, 95% CI: 0.48-0.82) (Table 5).

**Table 5:** ***Kappa*** **statistics of examiners with UE results**

UE		Examiner2					
		Score1	Score2	Score3	Score4	Score5	Total
	Score1	0	0	0	0	0	0
	Score2	0	8	1	0	0	9
	Score3	0	0	6	1	0	7
**Examiner1**	Score4	0	0	4	17	5	26
	Score5	0	0	0	0	4	4
	Total	0	8	11	18	9	46

*κ* = 0.65 (95% CI: 0.48-0.82), indicating the inter-examiner reliability reached a substantial agreement.

Based on the result, lesions with a score of 3, 4, and 5 were not very consistent between examiner 1 and examiner 2. To avoid discrepancy, two examiners had another check together and eventually reached an agreement on the results (Table 3). The results were further compared with pathological findings. The inter-rater reliability demonstrated a substantial agreement (*κ* = 0.77, 95% CI: 0.58-0.96) (Table 4).

### ABVS combined with UE

On the basis of complementary advantages, after the interpretation of ABVS and UE images were completed, the information of ABVS image and UE image were provided for the two examiners to make comprehensive assessment of all lesions. They redefined the category of ABVS and the score of UE of every lesion and identified the nature of lesions with both the morphological and the stiffness details. Therefore, the diagnosed accuracy would be increased (Table 3). The final results were compared with pathological findings (Table 4), which reached a perfect agreement (*κ* = 0.90, 95% CI: 0.77-1.00).

### Pathology

**Figure 1:**
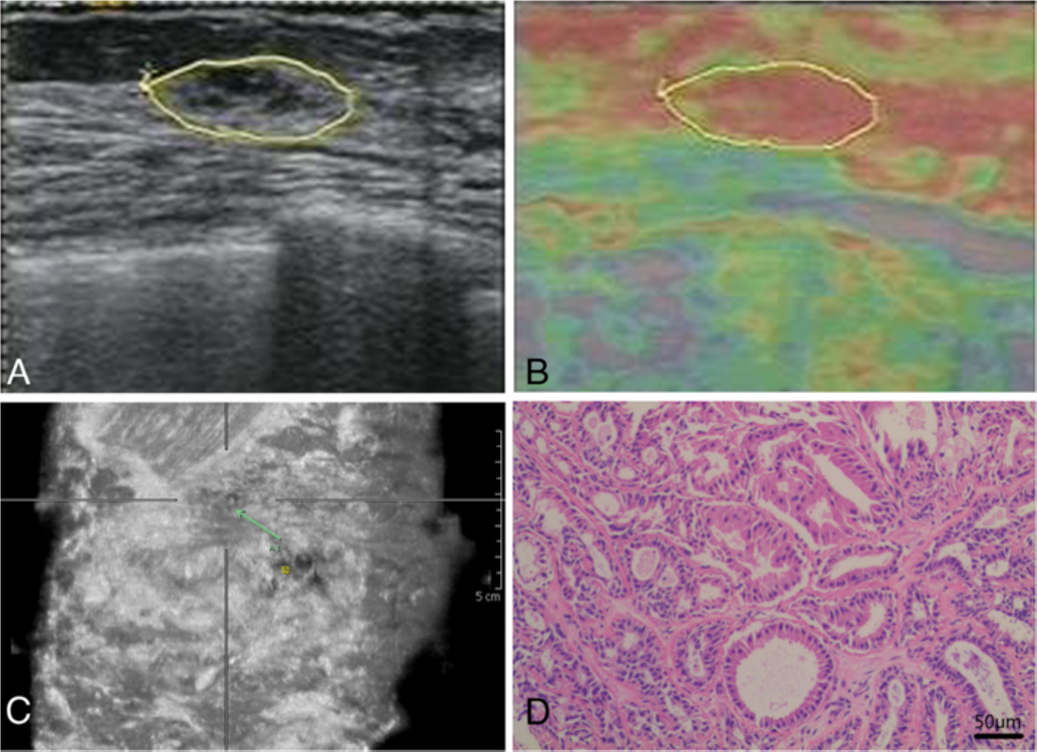
**US, UE, ABVS and histologic section image in a 51-year-old woman with intraductal papilloma. (A)** US image reveals hypoecho mass with cystic components and microcalcification. **(B)** ABVS image reveals mass with microcalcifications within the duct (arrow), which was misdiagnosed as malignancy. **(C)** UE image reveals almost entire lesion as red, indicating a hard lesion with UE score of 5. **(D)** Hemotoxylin and eosin (H&E) image reveals intact ductal lining with papillary structures (original magnification, × 200). US: Ultrasonography. ABVS: Automated breast volume scanner. UE: US elastography.

**Figure 2:**
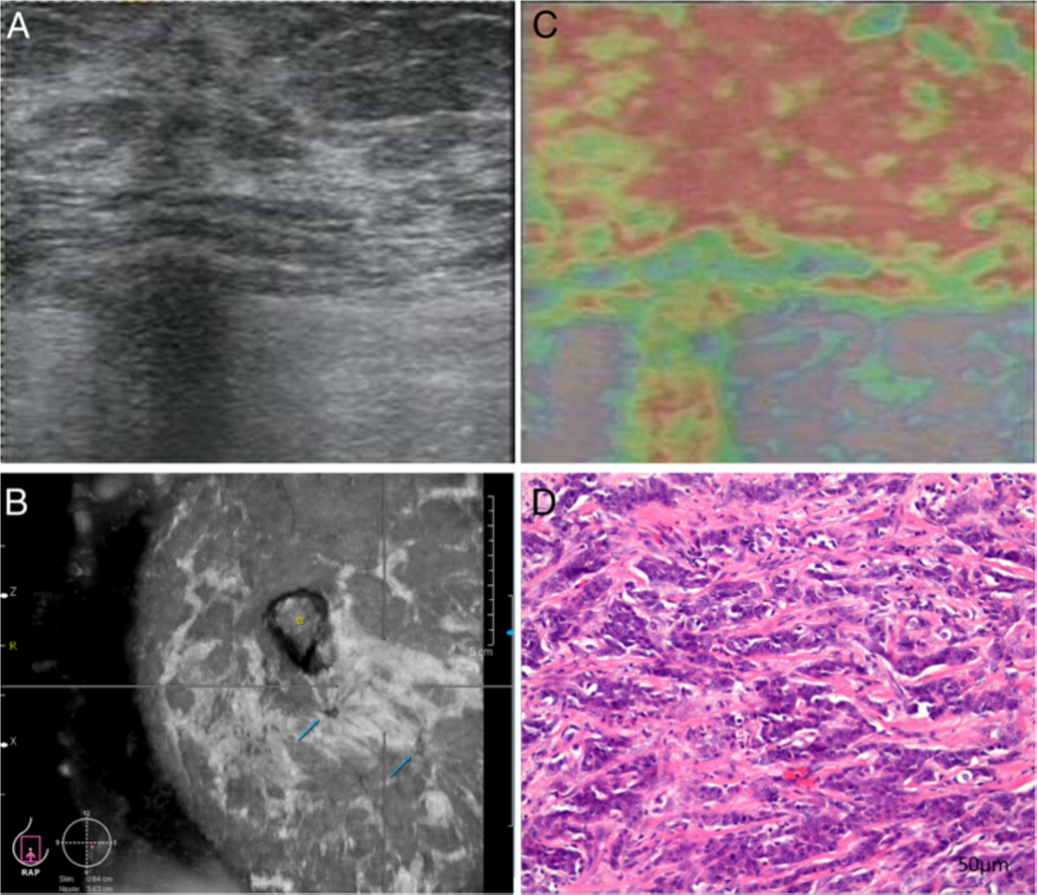
**US, UE, ABVS and histologic section image in a 41-year-old woman with invasive ductal carcinoma. (A)** US image shows nonpalpable lesion. **(B)** ABVS image shows retraction phenomenon as “crater” sign (arrow). **(C)** UE image shows entire lesion as red, indicating a hard lesion with UE score of 5. **(D)** Hemotoxylin and eosin (H&E) image shows microstructure in tumor including cytoplasmic and nuclear vacuolation, clumping of nuclear chromatin (original magnification, × 200). US: Ultrasonography. ABVS: Automated breast volume scanner. UE: US elastography.

All the lesions were biopsied using the 16G core needle after the patients signed the consented form. Pathological findings determined that 18 benign lesions were 6 mammary dysplasia, 8 fibroadenoma, and 4 intraductal papilloma (Figure 1) and that 28 malignant lesions consisted of 27 invasive ductal carcinoma (Figure 2) and 1 invasive cribriform carcinoma (Table 3).

### Diagnosis performance

**Figure 3:**
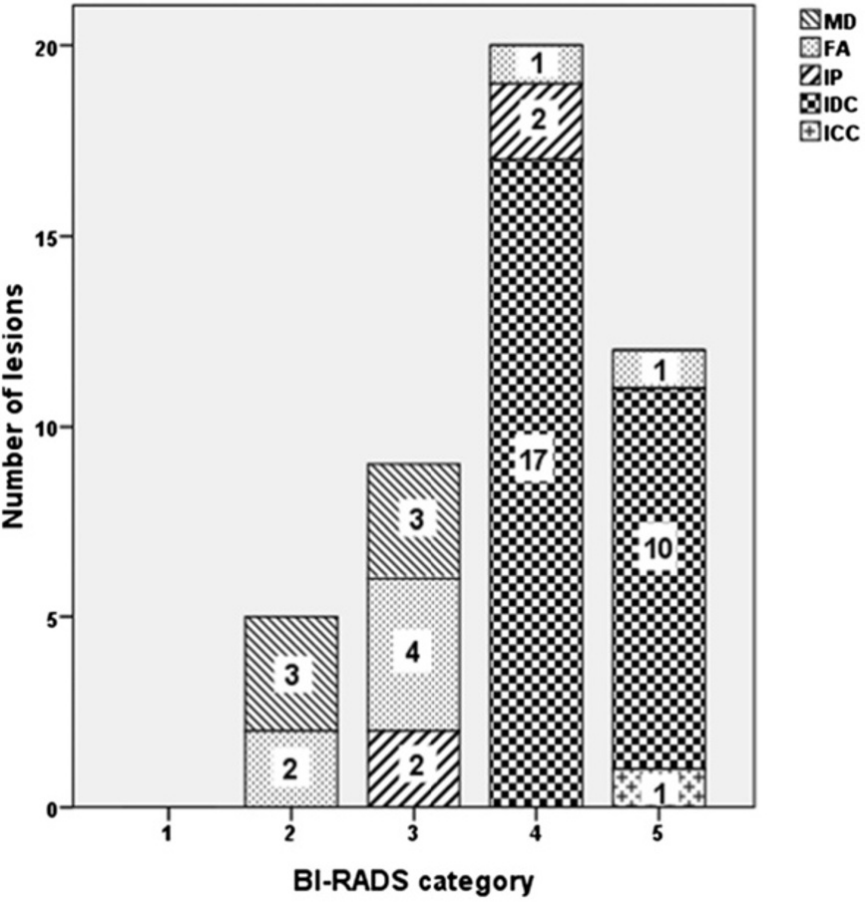
**Categories of ABVS and pathological findings.** Numbers in the chart represent the number of lesions. 4 lesions were misdiagnosed. ABVS: Automated breast volume scanner. MD: mammary dysplasia. FA: fibroadenoma. IP: intraductal papilloma. IDC: invasive ductal carcinoma. ICC: invasive cribriform carcinoma.

**Figure 4:**
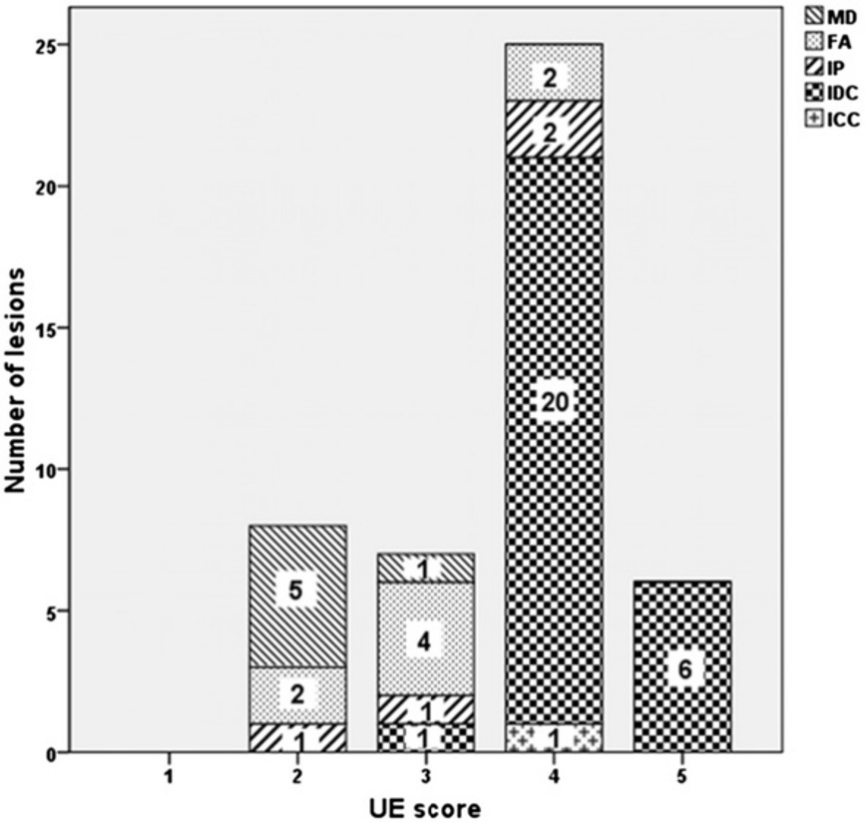
**Scores of UE and pathological findings.** Numbers in the chart represent the number of lesions. 4 lesions were misdiagnosed. UE: US elastography MD: mammary dysplasia. FA:fibroadenoma. IP:intraductal papilloma. IDC: invasive ductal carcinoma. ICC: invasive cribriform carcinoma.

**Figure 5:**
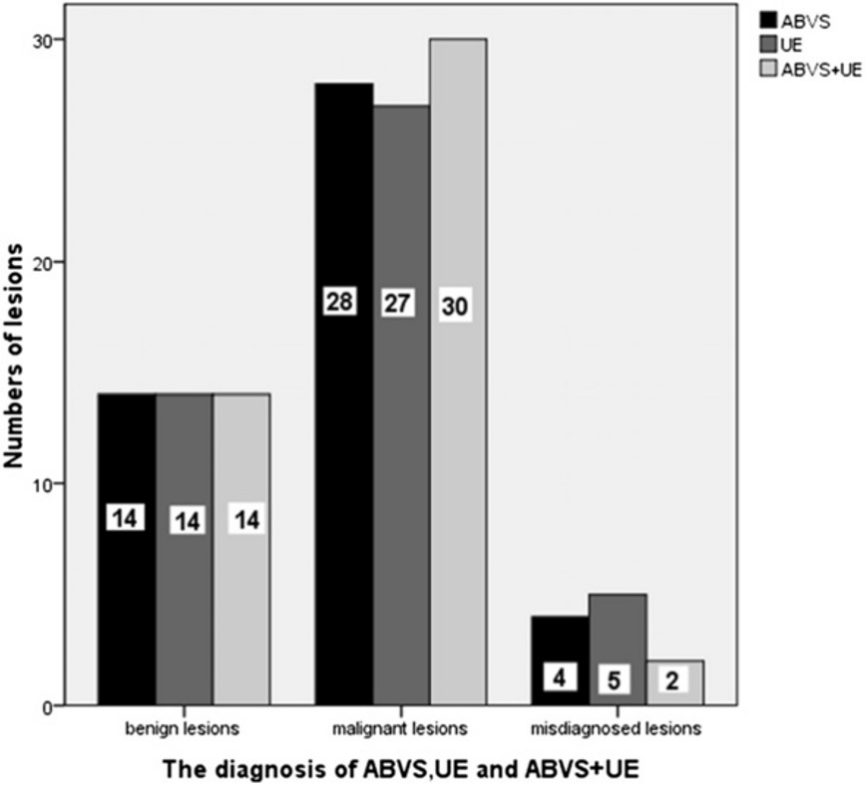
**The numbers of lesions correctly diagnosed and misdiagnosed by ABVS, UE, and ABVS + UE.** There were 4 lesions misdiagnosed by both ABVS and UE, 2 lesions misdiagnosed by ABVS + UE. ABVS: Automated breast volume scanner. UE: US elastography.

Compare BI-RADS category with pathological results, there were 14 (14/18) benign lesions consistent with pathological results and the malignant lesions were 28 (32/28), 4 benign lesions were misdiagnosed as malignant lesions (Figure 3). As respect to the UE results, 14 benign lesions and 27 malignant lesions were correctly diagnosed while 1 malignant lesion was misdiagnosed as benign and 4 benign lesions misdiagnosed as malignant (Figure 4). When ABVS and UE combined, only two benign lesions were misdiagnosed as malignant lesions (Figure 5). According to these results, the accuracy, sensitivity, specificity, PPN and NPN of ABVS, UE, and ABVS combined with UE were calculated. Though there were no statistically significance in any of the diagnostic performance index among these three methods, the accuracy, sensitivity, specificity, PPN and NPN for ABVS combined with UE were 95.7% (95% CI: 84.0-99.2), 100% (95% CI: 85.9-100), 87.5% (95% CI: 60.4-97.8), 93.8% (95% CI: 77.8-98.9), 100% (95% CI: 73.2-100), for UE were 89.1% (95% CI: 75.6-95.9), 96.4% (95% CI: 79.8-99.8), 77.8% (95% CI: 51.9-92.6), 87.1% (95% CI: 69.2-95.8), 93.3% (95% CI: 66.0-99.7), for ABVS were 91.3% (95% CI: 78.3-97.2), 100% (95% CI: 85.0-1.00), 77.8% (95% CI: 51.9-92.6), 87.5% (95% CI: 70.1-95.9), 100% (95% CI: 73.2-100), respectively, suggesting that the diagnostic performance of ABVS combined with UE was better than, or at least equal to, that of ABVS or UE alone (Table 6).

**Table 6:** **Diagnostic performance of ABVS, UE, and ABVS + UE**

Examina-tion	Accuracy	Sensitivity	Specificity	PPN	NPN
	95% CI	95% CI	95% CI	95% CI	95% CI
	(***P*** = 0.97)	(***P*** = 0.99)	(***P*** = 0.97)	(***P*** = 0.98)	(***P*** = 0.99)
**ABVS**	91.3%	100%	77.8%	87.5%	100%
	78.3-97.2	85.0-100	51.9-92.6	70.1-95.9	73.2-1.00
**UE**	89.1%	96.4%	77.8%	87.1%	93.3%
	75.6-95.9	79.8-99.8	51.9-92.6	69.2-95.8	66.0-99.7
**ABVS + UE**	95.7%	100%	87.50%	93.8%	100%
	84.0-99.2	85.9-100	60.4-97.8	77.8-98.9	73.2-1.00

The diagnostic performance of ABVS, UE and ABVS + UE was evaluated.

## Discussion

### Combined use of ABVS and UE for breast lesions is feasible

To our knowledge, this is the first report on combined use of ABVS and UE for evaluation of benign and malignant lesions of breast. The ability of ABVS to image large benign and malignant lesions automatically and UE to determine lesions stiffness effectively led to the assumption that the combination of these two methods is capable of detecting clinically occult benignancy and malignancy. This implies that the combination of ABVS and UE seems to be a promising tool to overcome the short comes of HHUS, ABVS, or UE when used alone, though its agreement rate and the diagnostic performance are in small increments. We propose that this may be because ABVS is inability to immediately adjust the modifying factors such as compression, the orientation of the probe, and the machine’s setting while acquiring the image in real-time when exploring further a questionable lesion. Though HHUS and UE may compensate these shortcomings, HHUS is lacking of standardization in diagnostic for the poor reproducibility of images and high variability of operators. And UE is not specific enough to diagnose lesions in morphology. On the other hand, both ABVS and UE are inability to perform color or spectral Doppler for tissue or lesion vascularity. Therefore, the combination of ABVS and UE provides minimal benefit to diagnostic performance.

### Excellent reproducibility

From a methodological point of view, agreement rate is an indicator for a new, experimental diagnostic method [11]. According to our results, ABVS and UE both displayed substantial inter-examiner agreement (ABVS: *κ* = 0.62, UE: *κ* = 0.65). The agreement among ABVS, UE, and ABVS combined with UE with pathological results were substantial or perfect (ABVS: *κ* = 0.81; UE: *κ* = 0.77; ABVS + UE: *κ* = 0.90). Deservedly, ABVS combined with UE showed a modest increase. Our results are consistent with literatures showing an acceptable range inter-examiner agreement of 0.18-0.80 on ABVS [12, 13, 15] and 0.48-0.75 on UE [20, 22, 26]. These variation ranges may be accounted for reproducibility of each method, characteristic of lesions and experience of examiners. ABVS is known for its ability to reconstruct and preserve high resolution and real-time images simultaneously, and then to playback the image data in the system, which corroborates an excellent reproducibility. However, examiners’ experiences and lesions’ features are uncontrollable factors. The senior ultrasound doctors are more sensitive to lesions and far more likely to make the correct diagnosis than the junior doctors, especially in regards to the benign lesions with small diameter and limited specificity [13]. On the other hand, UE, which has a simpler scoring system as BI-RADS category, demonstrated a slightly increased reliability of inter-examiners than ABVS. However, elasticity images are vulnerable to mechanical properties, and elastographic scanning parameters including applied strain, transducer frequency, band width, and radiofrequency sampling rate. Furthermore, the thickness of the breast and the depth of the lesions play a decisive role on the quality of elasticity images, which could affect the diagnostic performance significantly. While the thicker breast and deeper lesions produce low quality images and the less thickness breast and shallower lesions produce higher image quality [26, 27]. Our data suggest that ABVS combined with UE is a more practically useful method for diagnosis. We speculated that doctors with more than 10 years experience, patients (Asian women) with small chest, lesions with palpable consistency, a simple scoring system and advanced equipment were the major factors for improving the quality of images and minimizing the variability of inter-examiner to ensure the diagnostic performance.

### High diagnosis performance

With the good reproducibility and high agreement rate, ABVS, UE, and ABVS combined with UE are proven to have high diagnosis performance of detecting breast lesions and differentiating benign from malignant lesions. Significant amount of literatures have validated that ABVS has good diagnostic performance with accuracy of 66%-97%, specificity of 52.8%-95%, sensitivity of 82%-100% [11–17] and UE with sensitivity of 78.0%-100%, specificity of 21.0%-98.5% [19, 20]. Our study showed that diagnostic performance of ABVS combined with UE (95.7% accuracy, 100% sensitivity, 87.5% specificity, 93.8% PPV, 100% NPN) was slightly higher than UE (89.1% accuracy, 96.4% sensitivity, 77.8% specificity, 87.1% PPV, 93.3% NPN) or ABVS (91.3% accuracy, 100% sensitivity, 77.8% specificity, 87.5% PPV, 100% NPN) alone. Though there was no statistical significance between ABVS combined with UE and ABVS or UE alone, ABVS combined with UE was favorable to improve the diagnostic performance.

### Limitations

There are several limitations of our study. The first is the lacking of comparison with HHUS. According to the data reported, the diagnosis performance of ABVS or UE was better than, or at least equal to, that of HHUS [11–17, 19, 20]. These were great results convinced us that ABVS or UE would be a practical method in detecting breast lesions even HHUS was not being used. In fact, ABVS and UE are simple and convenient methods with their striking practical advantages of time saving, less technical training, low variability and high reproducibility. Unsurprisingly, we got the results as expected and consistent with the literatures. However, the experimental design would be improved if we compared them with HHUS. Second, the sample size was small. Among 46 lesions, there was no ductal carcinoma in situ (DCSI). Undoubtedly, microcalcifications would mostly appear on DCSI. The high resolution image of ABVS can provide better demonstration of breast anatomy and proper orientation. It makes it possible for identifying microcalcifications of ductal carcinoma in situ (DCSI). If we had samples of DCSI, our results might have varied. Third, we didn’t use Color Doppler ultrasound to analyze the vascularity of the lesions. This is able to provide blood supply and resistant index for identifying benign or malignant lesions. We didn’t apply strain ratio to evaluate the lesion stiffness either, which could be used as an objective and constant characteristic regardless of data acquisition or interpretation variability [28]. In addition, strain ratio can determine whether a lesion is benign or malignant [29]. In our study, two benign lesions (one was fibroadenoma, another was intraductal papilloma (Figure 1)) both with little spiculated margin on ABVS and almost the entire lesion in red on UE, we misinterpreted as malignancy. According to report [30], if the diameter of fibroadenoma was large, it may manifest itself with irregular lobulation, speculation and foliar margins. It is difficult to distinguish benign from malignant lesions. In this situation, we recommend a needle aspiration or excision biopsy for a histologic diagnosis. Referring to intraductal papilloma, it shows a diversity of histopathological features usually accompanied by intraductal hyperplasia, atypical ductal hyperplasia, DCIS, and even IDC. This led to variation of clinical features. Therefore, with these two benign lesions’ indeterminate characteristics, diagnosis is difficult and shows no sign of becoming easier [31]. It is not surprising that we made a misdiagnosis. However, if Color Doppler ultrasound and strain ratio could be used to conduct further analysis and review, more information would be available. Unfortunately, lack of the evaluation of vascularity is what the shortcoming of ABVS and UE [9].

## Conclusions

In conclusion, the results of our study show that both ABVS and UE demonstrated substantial inter-examiner reliability. With the advantages of good reproducibility, low variability, less operator training, time saving of ABVS, and the strength of simpler scoring system and operation procedure of UE, and combining these two methods would be favorable to improve diagnostic accuracy and specificity.

## Abbreviations

ABVS: Automated breast volume scanner
UE: US elastography
CI: Confidential interval
PPN: Positive predictive value
NPN: Negative predictive value
US: Ultrasonography
HHUS: Handheld ultrasonography
MD: Mammary dysplasia
FA: Fibroadenoma
IP: Intraductal papilloma
IDC: Invasive ductal carcinoma
ICC: Invasive cribriform carcinoma
DCSI: Ductal carcinoma in situ.
